# The General Medical Council

**Published:** 1904-12-03

**Authors:** 


### The Hospital.

A JOURNAL OF

The Medical Sciences and Hospital Administration.

VOL. XXXVII.—No. 949. DECEMBER 3, 1904.

### THE GENERAL MEDICAL COUNCIL.

THE eightieth session of the General Medical Council, which was concluded last Tuesday, will be memorable as the last during which the presidential chair was filled by Sir William Turner, who announced in his opening address that, although the period for which he had been elected, both as representative of the University of Edinburgh and as president of the Council, would not expire until the close of 1906, he had come to the conclusion that the onerous nature of his duties as Principal of the University rendered him unable to retain the presidential chair. He announced at the same time his intention to present that emblem of authority—a mace—to the Council as a memento of the six years during which he had presided over its deliberations, and requested that his successor might be elected before the termination of the session. Sir William's tenure of office has been in every respect worthy of himself and of the great University which he represents, and has been marked by unruffled courtesy, unfailing dignity, and by a certain serenity of wisdom which has kept within bounds the more intemperate spirits among his colleagues, while, at the same time, it has never afforded to them the vantage ground of a grievance. He was entertained by the Council at a farewell dinner on Saturday, and will be long remembered and regretted in the Council chamber. At the conclusion of the session last Tuesday Dr. Donald MacAlister was unanimously elected to the chair in succession to Sir William Turner.

The struggle between the General Medical Council and the various licensing bodies, with regard to the control of the preliminary stages of medical education, has to some extent entered upon a new phase, and has given rise to pretensions on the part of some of the latter which seem to emphasise the desirability of committing the matters in dispute to a single authority rather than to 19 or 20 separate ones. At its last session the Council passed a resolution requesting the Privy Council to introduce into Parliament a Bill to confer upon the Council statutory powers to establish and maintain registers of medical and dental students, and to impose a fee not exceeding £1 for registration therein. Such a Bill would also, of course, confer upon the Medical Council the power to determine the conditions of registration, and would thus permit them to require whatever evidence of sufficient preliminary education they might deem expedient. The Royal Colleges in London, and the University of Oxford, were prompt to take alarm, and appealed to the Privy Council for the protection of their respective rights, with the result that the Medical Council was officially advised to confer with these bodies, and to endeavour to establish a *modus vivendi*. Considering that the point at issue is fundamental, the Medical Council desiring to exercise complete control over medical education, and to enforce the maintenance of a definite minimum standard, while the licensing bodies desire to retain complete independence, it is tolerably evident that no conference is likely to lead to agreement; and the Medical Council was probably well advised in dismissing the suggestion as an idle one. After various resolutions and amendments had been discussed, it was finally resolved that a reply be sent to the Privy Council stating that the Medical Council now sees great difficulties from opposition by certain licensing bodies, but that it will be prepared, at the proper time, to offer an alternative proposition which it hopes may be approved of by the Lord President. "When I says as you were," explained a drill-sergeant to a squad of Volunteers, "I means as you wos," and this appears to be the precise position assumed by the Medical Council. It would not be satisfactory to the public if the public understood it, and knew the importance of the issues which are at stake; but, as they are never likely to do either one or the other, it is possible that the existing deadlock may continue for an indefinite period. As a matter of common sense it is evident that the Legislature intended the Council to control medical education, and that it failed to confer the powers necessary for the purpose. As a natural result—

> Chaos umpire reigns,
> And by decision more embroils the fray.

In the exercise of its powers to punish professional delinquency, the Council has removed from the Register the names of two medical practitioners, one of whom was recently convicted of obtaining money under false pretences, while the other had been proceeded against in connection with a divorce case which was recently decided.

Other important business before the Council was the consideration of a report by the Medical Com panies' Bill Committee. The report expressed satisfaction at the fact that the legal advisers of the Board of Trade concurred with the judgment of Chief Baron Palles, and held that titles which were calculated to deceive the public into supposing that they would receive treatment by persons possessed of special skill in medicine or in dentistry would disentitle the company to registration. This is a step in the right direction, but it is at best a very partial remedy for the evils arising from company incorporation, chief among which is the avoidance of any personal responsibility. As the report pointed out, "the servants of such a company (medical or dental) might be, and had been, by means of lectures, pamphlets, advertisements, etc., placed in such a position that the public were led to suppose that they would receive skilled medical or dental treatment at their hands, and they might display ignorance or commit malpractice, and even be censured by a coroner, without any legal remedy being available against them. They were the irresponsible agents of an irresponsible company." When the General Medical Council has been able to attract the active attention of our administrators to the very obvious flaw which this statement reveals in the legal code, it will have supplied an emphatic answer to those persons who complain that the Council does not sufficiently fulfil its duty of protecting the public from fraud and injury perpetrated in the name of medicine.

